# Nondestructive Evaluation of Composite Bonding Structure Used in Electrical Insulation Based on Active Infrared Thermography

**DOI:** 10.3390/polym14163373

**Published:** 2022-08-18

**Authors:** Chenjun Guo, Lishuai Liu, Hongwei Mei, Yanxin Tu, Liming Wang

**Affiliations:** 1 Kunming Power Supply Company of China Southern Power Grid Company, Kunming 650011, China; 2 Engineering Laboratory of Power Equipment Reliability in Complicated Coastal Environments, Tsinghua Shenzhen International Graduate School, Tsinghua University, Shenzhen 518055, China; 3 Key Laboratory of Pressure Systems and Safety of MOE, School of Mechanical and Power Engineering, East China University of Science and Technology, Shanghai 200237, China

**Keywords:** active infrared thermography, live detection, electrical equipment, composite insulation, silicone rubber, fiber reinforced plastic, differential thermal image, frame difference thermal image

## Abstract

Nondestructive testing and evaluation of composite insulating components of electrical equipment is extremely necessary for assuring the safety of a power system. However, most existing nondestructive testing methods are not applicable for fast and effective live detection due to their time-consuming operation, high cost, and contact or near-field measurement. In this work, the effectiveness of active infrared thermography was investigated for detecting defects in silicone rubber (SIR)–fiber-reinforced plastic (FRP) bonding structures, which have been commonly used in insulating components of power equipment. The effectiveness of differential thermal image for enhancing the contrast of defective and sound areas and eliminating additive noise was demonstrated. Particularly, frame difference thermal image obtained by subtracting two differential thermal images extracting from respectively before and after the contrast inversion was proposed to enhance defect identification. The results revealed that defects of various sizes and depths such as voids, cracks, and interface disbonding of the SIR–FRP bonding structure were accurately detected by thermographic data. With the advantages of a quick and simple process, safety, universal applicability, visual results, far-field measurement, and quantitative defect estimation capabilities, active infrared thermography would be quite promising for live detection of electrical equipment.

## 1. Introduction

Electrical equipment in operation is subjected to the combined action of electrical, thermal, mechanical, and chemical factors, which results in insulation deterioration, equipment failure, and power outrages [1]. The insulating property has proven to be the key to ensuring normal operation and extending service life of electrical equipment [2]. Consequently, the technology of insulation detection of power apparatus is extremely necessary for assuring the safety of power system. Composite insulating materials are increasingly used in electrical equipment owing to their favorable dielectric and mechanical properties, but meanwhile are prone to age and generating defects. In recent years, a large amount of research work has emerged in this field, and some useful methods for defect detection of composite insulating materials have been proposed. Terahertz time-domain spectroscopy (THz-TDS) technology utilizes pulsed waves ranging from 0.02 to 2 THz to detect defects of composite insulators, and the system can measure distances up to 15 cm [3]. Microwave can detect internal defects such as voids, cracks, and small metal inclusions in composite insulators according to the different dielectric constants corresponding to different types of internal defects [4,5]. Ultrasonic sensors are usually deployed to detect insulation conditions of various power equipment such as partial discharges in transformer oil, deterioration of generator stator insulation, and internal defects in composite insulators [6,7,8]. X-ray inspection can identify the location and size of defects according to the radiation imaging with different attenuation degrees transmitted by the inspected structure and is widely used in external diagnosis of power transmission and distribution equipment [9,10]. Acoustic emission detection is a technology that realizes detection by receiving and analyzing the acoustic signal emitted by the equipment. The acoustic emission signal used in this method is the transient elastic wave caused by the rapid release of energy caused by local defects in the material, and thus this technique has been used for detection, measurement, and location of partial discharges and material degradation in power appliances [11,12]. The above methods all play an important role in quality assurance in the manufacturing process and offline inspection, but still have limitations such as time-consuming operation, high cost, and contact measurement. Most of the methods are often not suitable for live detection of electrical equipment, where the measurement distance is required to be long enough to ensure insulation safety. Since the timeliness, convenience, and economy of offline inspection, which needs power-off, is actually less than satisfactory, it is necessary to explore a novel method for detection and evaluation of electrical equipment defects, which meets the requirements of simple operation, wide applicability, and far-field measurement.

**Figure 1:**
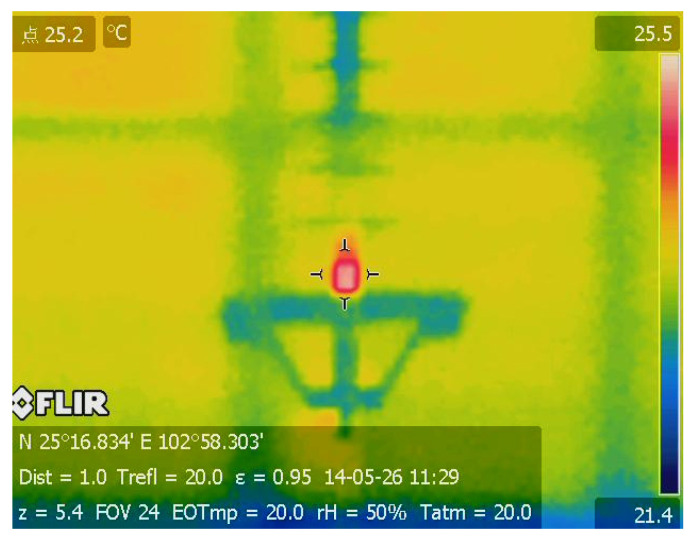
Infrared thermographic inspection of abnormal temperature rises at high-voltage end of onsite composite insulator.

Infrared thermography is a technique for converting infrared energy into electronic signal to produce the temperature distribution of inspected object by non-contact thermal imaging devices [13,14,15]. Owing to its advantages of safety, universal applicability, far-field measurement, large inspection coverage, and being free from electromagnetic interference, infrared thermography has been successfully used for condition monitoring and fault diagnosis of power equipment over decades [16,17,18]. Various kinds of electrical equipment faults, such as loose connection, corrosion, short and open circuits, aging, harmonics influence, internal defects, load imbalance, and overloads, manifest as an abnormal temperature rise, which in turn accelerates the deterioration of equipment. Thus, infrared thermal inspection is a preferred option for preventive and predictive maintenance of electrical components with the abilities of instantly identifying and locating the hot spots and classifying the severity of the problem. A typical infrared thermographic result for defect inspection is provided in Figure 1, which shows an abnormal temperature rise at the high-voltage end of onsite composite insulator. It has been demonstrated that the local heating phenomenon of composite insulator is closely related to internal partial discharge, moisture dielectric polarization, and insulation resistance deterioration of materials [19]. Therefore, it is reasonable to infer that this composite insulator has potential danger of fracture accident and should be replaced at once.

Although advanced infrared detector ensures the superiority of infrared thermographic detection in quick, accurate, large-scale, and long-distance signal acquisition compared with other sensor-based detecting techniques, abnormal heating is not a perfect indicator of all electrical failures in power systems. There are many reasons for electrical equipment heating and the correspondences between abnormal heating and equipment failure are still ambiguous and unreliable [20]. Even if the heating caused by internal defects in the equipment can be detected, it is difficult to identify the causes of heating, i.e., the specific equipment failure, and model the quantitative relationship between the defect characteristics and the heating phenomenon [21]. Moreover, sometimes the temperature of facilities inspected do not increase even though the fault indeed exists.

Active infrared thermography (AIT) is a novel nondestructive testing and evaluation (NDT&E) technique which has been developing rapidly in recent years. The proposed AIT detection method effectively solves the difficulties faced by the existing passive infrared thermography detection technology. By applying a controllable thermal excitation to the tested specimen, a changing temperature field is generated inside the tested specimen, and then the time-series dynamic temperature distribution on its surface is observed, and the abnormal internal structure of the specimen can be analyzed from it [22,23]. Different from the totally passive thermal information receiving process of abovementioned passive infrared thermography, the active thermography can be regarded as a two-port network. Based on the theory of heat transfer, there is a definite correspondence between the internal structural characteristics of the specimen, the excitation function, and the temperature change of the specimen surface. Therefore, the AIT method has strong stability and reliability, and can be used for quantitative detection of defects. It is noted that the detection distance of AIT is limited by the effective heating distance of external heating excitation and cannot reach the level of passive infrared thermography detection. However, with the further upgrade of the excitation source, AIT still has the prospect of field application for live detection. At the same time, compared with other existing offline nondestructive testing methods, the AIT method has the advantages of non-contact measurement, fast detection speed, and intuitive results. Liu et al. [24] proposed a semi-supervised method based on AIT for the measurement of surface pollution severity of porcelain plate. Zhang et al. [25] detected interfacial defects with the width of 1 mm below 2 mm thick silicon coating based on transient thermal wave. J. Moran and Nik Rajic [26] used dynamic pulse phase thermography to detect the composite impact damage based on AIT. However, the defects in the composite insulating usually occurred in the interface of bonding structure. Previous studies of AIT focused on surface defects in composites, and the detection accuracy of inner defects was poor. The detection effect of AIT can be improved by using the enhancement algorithm [27]. Therefore, the AIT method has great application potential for defect detection in the production process of composite insulating components and even in the operation process.

This article presents the investigation of AIT for defects detection of silicone rubber (SIR) –fiber-reinforced plastic (FRP) bonding structures, which are widely used for insulating components of electrical equipment. Defects of various sizes and depths such as voids, cracks, and interface disbonding were tested and identified by thermographic analysis. The effectiveness of differential thermal image for enhancing the contrast of defective and sound areas and eliminating additive noise was demonstrated. In addition, frame difference thermal imaging obtained by subtracting two differential thermal images extracting from respectively before and after the contrast inversion was proposed to enhance defect identification.

## 2. Methodology

### 2.1. Active Infrared Thermography

**Figure 2:**
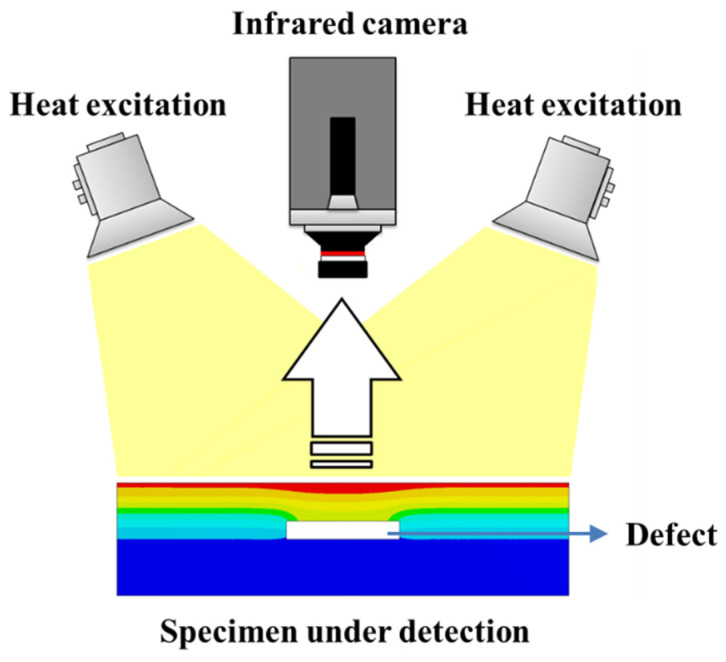
The principle of pulsed infrared thermography detection.

Pulsed thermography (PT) employed in this work is one of the simplest, fastest, and most developed AIT techniques. The principle of nondestructive testing process based on pulsed infrared thermography is illustrated in Figure 2. External thermal excitation sources are used to apply pulsed heat flow to the tested specimen, and at the same time, an infrared thermal imager is used to record the changing temperature field of the tested specimen surface. Due to the different thermophysical properties of the defective area and the non-defective area inside the tested specimen, according to the theory of heat transfer, the heat flow spreads non-uniformly inside the specimen, resulting in the surface temperature evolution corresponding to the defective and the non-defective area shows differences. By processing and analyzing the infrared heat map sequence, it is possible to determine the existence of defects and obtain information such as defect location, size, depth, etc. [28,29]. According to Fourier’s law, the thermal conductivity differential equation shown in Formula (1) can be established, and the whole physical process of detection can be regarded as solving the thermal conductivity differential equation after determining the boundary conditions [30]:

(1) $$\nabla^{2}T-\frac{1}{\alpha }\cdot \frac{\partial T}{\partial t}\;=\;0$$

where $\nabla^{2}$ is Laplace operator; *T* is temperature evolution of the sample; α= λ/(ρc) is the thermal diffusivity; *λ* is the thermal conductivity; *ρ* is the density; *c* is the specific heat; and *t* is time.

The heat pulse excitation can be expressed as

(2) $$q=q_{0}\delta (x)\delta (t)$$

where *δ*(*x*) and *δ*(*t*) are Dirac functions, which means

(3) $$\int_{-\infty }^{+\infty }\delta (x)=1,\;\mathrm{and}\;\mathrm{when}\;x\neq 0,\;\delta (x)=0$$

Solve (1) with initial conditions

(4) $$T(x,0)=T_{0}$$

and boundary conditions

(5) $${-\lambda \frac{\partial T}{\partial x}|}_{x=0}={q|}_{x=0}=q_{0}\delta (t)$$

The solution can be obtained by Laplace transformation as

(6) $$T(x,t)=T_{0}+\frac{q_{0}}{\sqrt{\pi \rho c\lambda t}}e^{-\frac{x^{2}}{4\alpha t}}$$

The surface temperature of non-defective area changes over time as

(7) $$T_{sound}(0,t)=T_{0}+\frac{q_{0}}{\sqrt{\pi \rho c\lambda t}}$$

The heat flow will be reflected when encountering a defect, and reflection will happen again at the surface. The thermal wave reflects periodically until the final reflected wave attenuates to zero. The surface temperature of defective area above a defect with the depth of *h* can be obtained as

(8) $$T_{defect}(0,t)=T_{0}+\frac{q_{0}}{\sqrt{\pi \rho c\lambda t}}\left[1+\sum_{n=1}^{\infty }e^{-\frac{{\left(nh\right)}^{2}}{\alpha t}}\right]$$

The thermal contrast between defective and non-defective area is

(9) $$\Delta T=\frac{q_{0}}{\sqrt{\pi \rho c\lambda t}}\sum_{n=1}^{\infty }e^{-\frac{{\left(nh\right)}^{2}}{\alpha t}}$$

### 2.2. Data Fitting and Reconstruction

Different from other detection technologies such as passive infrared imaging detection, a distinctive feature of AIT is that the detection process collects continuous imaging signals, that is, an image sequence rather than a single frame of image, which also means that AIT describes dynamic rather than static information. This makes the AIT detection signal contain richer information describing the defects and makes it possible to mine and evaluate the defect state of the insulating material in multiple dimensions. Before defect identification and analysis, it is necessary to compress and reconstruct the image sequence to reduce the amount of data to be stored and the temporal inter-frame noises. Furthermore, the reconstructed image sequence is the basis for applying many advanced defect identification and quantification techniques in subsequent processing.

Equation (7) shows the temperature change on the surface of homogeneous isotropic materials. Taking the natural logarithm of both sides of (7) after transposition, then we can get a linear expression as

(10) $$\ln\left[T(t)-T_{0}\right]=\ln\left(\frac{q_{0}}{\sqrt{\pi \rho c\lambda }}\right)-\frac{1}{2}\ln t$$

Extract the temperature-time curve of a certain pixel in the transient thermal image sequence, subtract the temperature value of the pixel before excitation, take the natural logarithm, and plot the temperature difference-time logarithm curve with the natural logarithm of time. According to Formula (10), the temperature difference-time logarithmic curve of the defect-free area on the surface of the tested specimen should be a straight line with a slope of −1/2. In actual detection, the temperature difference-time logarithmic curve will deviate at the discontinuous interface. The deviation position has the depth information of the discontinuous interface, and the slope of the deviation has the thermophysical property information of the material on the other side of the discontinuous interface [31,32]. Therefore, the transient temperature variation of any pixel in the thermal image sequence can be fitted by a polynomial function as shown below.

(11) $$\ln\left[T(t)-T_{0}\right]=\sum_{n=0}^{N}a_{n}{\left(\ln t\right)}^{n}$$

Hence the acquired thermal data can be well fitted with a polynomial via least square method. Assuming data points $\left(x_{i},y_{i})\;(i=0,1,\cdots ,m\right)$ were obtained, it is hoped to find a polynomial function $p_{n}(x)=\sum_{k=0}^{m}a_{k}x^{k}$, which meets the following formula

(12) $$\arg\min\left\{I=\sum_{i=0}^{m}{\left[p_{n}(x_{i})-y_{i}\right]}^{2}={\sum_{i=0}^{m}\left(\sum_{k=0}^{n}a_{k}x_{i}^{k}-y_{i}\right)}^{2}\right\}$$

Obviously, it is needed is to find the extreme value point of the function $I=I(a_{0},a_{1},\cdots ,a_{n})$ by the necessary condition to seek the extreme value of multivariate function as

(13) $$\frac{\partial I}{\partial a_{j}}=2\sum_{i=0}^{m}\left(\sum_{k=0}^{n}a_{k}x_{i}^{k}-y_{i}\right)\cdot x_{i}^{j}=0,j=0,1,\cdots ,n$$

(14) $$\left[\begin{array}{cccc} m+1 & \sum_{i=0}^{m}x_{i} & \cdots & \sum_{i=0}^{m}x_{i}^{n}\\ \sum_{i=0}^{m}x_{i} & \sum_{i=0}^{m}x_{i}^{2} & \cdots & \sum_{i=0}^{m}x_{i}^{n+1}\\ \vdots & \vdots & & \vdots \\ \sum_{i=0}^{m}x_{i}^{n} & \sum_{i=0}^{m}x_{i}^{n+1} & \cdots & \sum_{i=0}^{m}x_{i}^{2n} \end{array}\right]\left[\begin{array}{c} a_{0}\\ a_{1}\\ \vdots \\ a_{n} \end{array}\right]=\left[\begin{array}{c} \sum_{i=0}^{m}y_{i}\\ \sum_{i=0}^{m}x_{i}y_{i}\\ \vdots \\ \sum_{i=0}^{m}x_{i}^{n}y_{i} \end{array}\right]$$

It can be certified that the coefficient matrix of (14) is a symmetric positive definite matrix and it has a unique solution $a_{k}(k=0,1,\cdots ,n)$. The fitted polynomial is given as

(15) $$p_{n}(x)=\sum_{k=0}^{m}a_{k}x^{k}$$

Then, the time variation of every pixel from thermal image sequence can be reconstructed according to

(16) $$T\left(t\right)=T_{0}+\exp\left[\sum_{n=0}^{N}a_{n}{\left(\ln t\right)}^{n}\right]$$

Therefore, regardless of the length of the image sequence, only the polynomial coefficients need to be saved. Data compression performance and operation speed are significantly improved. At the same time, different from the discrete temperature data of the thermal image sequence directly collected by the infrared thermal imager, the reconstructed temperature variation curve is differentiable.

### 2.3. Image Processing and Enhancement

In AIT, defects are identified via the thermal contrast between defective and sound area, which can be expressed as follows and the high order terms are negligible because of the high attenuation of thermal wave.

(17) $$\Delta T=\frac{q_{0}}{\sqrt{\pi \rho c\lambda t}}e^{-\frac{h^{2}}{\alpha t}}$$

The surface temperature difference rises first to a maximum and then descends gradually until becoming zero. It is hard to recognize the defects when the surface temperature difference is too small or the acquired thermal images have too much noise. Therefore, image enhancement technologies are necessary for better defect identification. Take derivative of (17) as follows

(18) $$\frac{d(\Delta T)}{dt}=\frac{q_{0}}{\sqrt{\pi \rho c\lambda }}e^{-\frac{h^{2}}{\alpha t}}\left(-\frac{1}{2}t^{-\frac{3}{2}}+\frac{h^{2}}{\alpha }t^{-\frac{5}{2}}\right)$$

(19) $$\frac{d^{2}(\Delta T)}{dt^{2}}=\frac{q_{0}}{\sqrt{\pi \rho c\lambda }}e^{-\frac{h^{2}}{\alpha t}}\left(\frac{3}{4}t^{-\frac{5}{2}}-\frac{3h^{2}}{\alpha }t^{-\frac{7}{2}}+\frac{h^{4}}{\alpha^{2}}t^{-\frac{9}{2}}\right)$$

**Figure 3:**
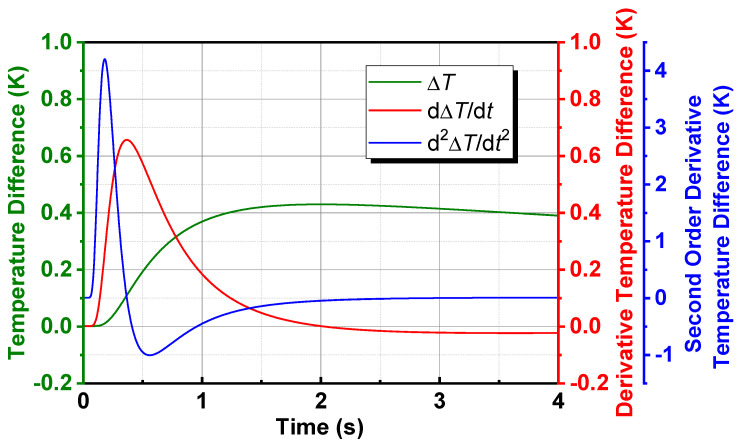
Temperature difference over time and its first and second derivative.

Then, the thermal contrast variation over time and its first and second derivative can be plotted in Figure 3. The coefficients were set to 1 for simplicity. It shows that the contrast between sound and defective areas is enhanced to a certain degree after differential processing. Furthermore, the additive noise can be also effective eliminated. Thus, the differential thermal image sequence, which is supposed to enhance the defect identification, can be generated through differential processing of the reconstructed temperature variation of every pixel.

It is clearly observed from Figure 3 that both the first and second order derivative of thermal contrast change from positive in the first stage to negative, and finally become zero. That is to say, in the first and second order differential thermal image sequence, the grayscale of defective area gradually increases to a certain extent, when it is always higher than that of sound area in this stage, and then begins to decrease until it is lower than that of sound area. After falling to a certain extent, it starts to rise again until the grayscale of whole image turns into the same. A frame difference thermal image (FDTI) is obtained by subtracting the two images extracting from before and after the inversion respectively, and the grayscale contrast and defect identification are well enhanced.

## 3. Materials and Methods

### 3.1. Samples

Silicone rubber–fiber-reinforced plastic bonding structures (SIR-FRP) are widely utilized in composite insulation of electrical equipment. This composite bonding structure is formed by bonding methyl vinyl silicone rubber and glass fiber-reinforced epoxy resin through an organosilicon coupling agent. In order to investigate the validity of PT for inspecting composite insulating materials, plate specimens containing the typical defects, including void, crack, and interface disbond, were prepared for the following inspection experiments. These three typical defects, i.e., voids in SIR, cracks in FRP, and disbond in SIR–FRP bonding structure, are experimentally studied in Section 4.1, Section 4.2, and Section 4.3, respectively.

**Figure 4:**
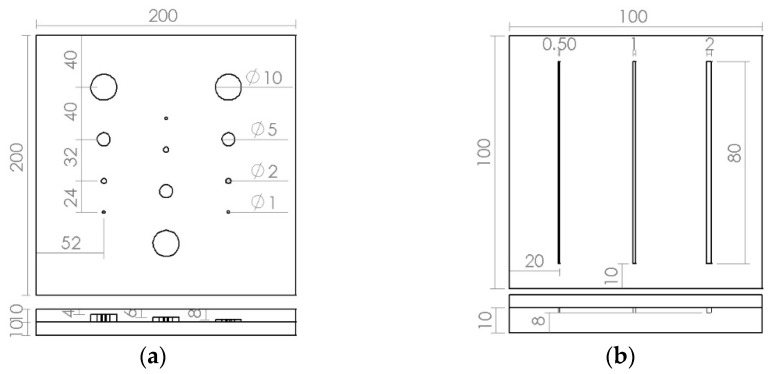
Design drawings of test specimens with artificial defects (unit: mm). (**a**) Voids in silicone rubber. (**b**) Cracks in fiber-reinforced plastic.

Figure 4 shows the main design parameters of SIR–FRP bonding test specimens with artificial defects. For specimen with void defects in SIR, the dimension of SIR and FRP plate specimen is 200 mm × 200 mm × 10 mm. The SIR plate consists of 12 flat-bottom holes, which are used for simulation of void defects of different sizes at different depths. The layout and dimension of flat-bottom holes are shown in Figure 4a. For illustration purpose, each void defect was marked with a combination of a letter and a number. The letters A, B, and C were used for distinguishing the depth of defect, and the numbers 1 to 4 were used for distinguishing the diameter. For example, A1 represented the void defect with the depth of 4 mm and the diameter of 10 mm, and C4 represented the one with the depth of 8 mm and the diameter of 1 mm. Figure 4b shows the bonding plate sample with three cracks in FRP part. The dimension of FRP plate is 100 mm × 100 mm × 10 mm. The SIR plate has various thicknesses of 1 mm, 3 mm, and 5 mm. Each crack is 80 mm long and 2 mm deep. The cracks were marked as CR1, CR2, and CR3 for their different widths of 2 mm, 1 mm, and 0.5 mm respectively. Furthermore, FRP plates with the size of 200 mm × 200 mm × 10 mm were bonded with two SIR sheets with the area of 200 mm × 100 mm to prepare disbond defects. During the bonding process, several parts of the interface were not bonded. In order to simulate disbond defects under different thickness, SIR sheets are available in various thicknesses of 2 mm, 3 mm, and 5 mm.

### 3.2. Experimental System

**Figure 5:**
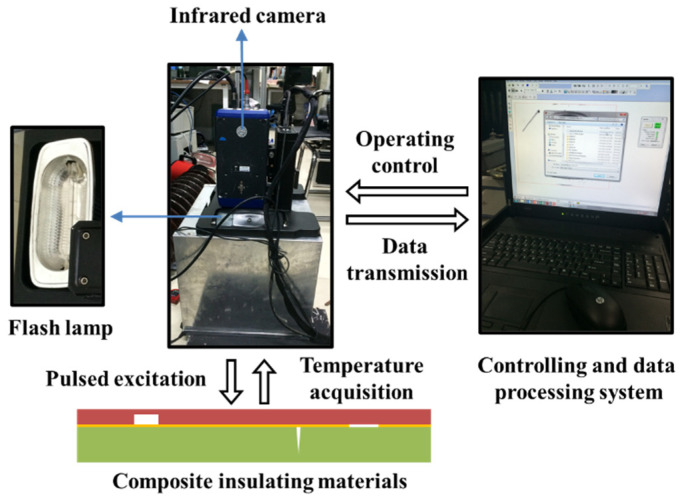
Experimental facilities and working principle.

The experimental platform is shown in Figure 5, which consists of a detection device and a control and data processing system. The detection device is equipped with two high-power modulated flash lamps with a light pulse width of 2 ms and a maximum energy of 9600 J. A FLIR SC7000 focal plane array long-wave infrared thermal imager is installed above the detection device. It adopts advanced cooled quantum well infrared photodetector technology and a highly reliable internal circulation refrigerator to reduce noise and provide high-resolution images. The working band of the thermal imager is 3.1~5.6 μm, and it can collect images with a resolution of 640 × 512 pixels. The built-in data acquisition interface of the control and data processing system is connected to the thermal imager to collect infrared thermal wave images and perform image processing and data analysis. The lower part of the control and data processing system charges the capacitor power supply for the flash excitation system and is connected with the detection device through the corresponding interface. In the experiment, the acquisition frequency of the thermal imager was set to 200 Hz and the acquisition time was set to 30 s.

## 4. Results and Discussion

In the experiments, thermal excitation should always be applied on the SIR side of the plate specimen to simulate the actual situation. After being heated by the flash pulse, the transient variation of the surface temperature of the plate sample is recorded by an infrared camera, and then displayed, stored, and processed in the form of thermal image sequence. After logarithm operation, polynomial fitting and image sequence reconstruction, differential operation of reconstructed thermal image sequence can be immediately done to produce more recognizable display of defects. Raw thermal images (RTI) and the first and second order differential thermal images (DTI) were presented for defect inspection in this work.

### 4.1. Detection of Voids in Silicone Rubber

**Figure 6:**
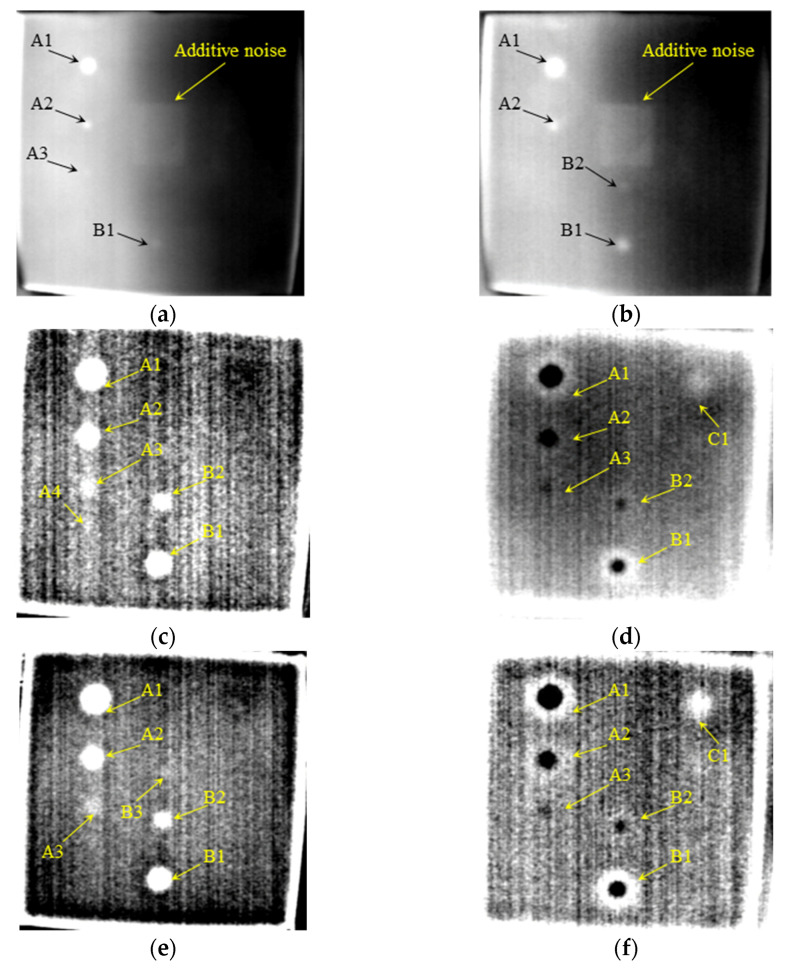
Detection results of voids in silicone rubber. (**a**) The 100th frame RTI. (**b**) The 300th frame RTI. (**c**) The 10th frame 1st DTI. (**d**) The 275th frame 1st DTI. (**e**) The 175th frame 2nd DTI. (**f**) The 450th frame 2nd DTI.

Figure 6 shows the AIT detection results of voids in silicone rubber. In Figure 6a, the 100th frame RTI (acquired at 0.5 s), only defects A1, A2, A3, and B1 can be observed. Defects B1 and B2 can be recognized in Figure 6b, the 300th frame RTI (acquired at 1.5 s), but A3 is not so clear in this frame. This phenomenon indicates that deeper defects appear later in the thermal image sequence because of the heat transfer process. In other words, the optimal time to identify a defect depends on its depth. Smaller or deeper defects cannot be found in RTIs. The square spot in the center of the image which could be seen in Figure 6a,b is the shadow of infrared camera lens. The temperature, which shows in thermal images as grayscale, of left part is higher than the right part of the plate owing to uneven heating. To decrease noise effects and enhance detection performance, DTIs were employed to enhance signal-to-noise ratio (SNR) and recognize the small defects better. It can be observed that additive noise (the shadow of infrared camera lens) and uneven heating were truly eliminated in the first and second order DTIs shown in Figure 6c–f.

The SNR quantifies the relationship between the defect’s visibility in the image and the level of background noise. The SNR is calculated as follows:

(20) $$SNR=20\cdot \log_{10}\frac{\left|\frac{1}{N_{D}}\sum_{i=1}^{N_{D}}I_{D}^{\left(i\right)}-\frac{1}{N_{S}}\sum_{j=1}^{N_{S}}I_{S}^{\left(j\right)}\right|}{\sqrt{\frac{1}{N_{S}}\sum_{j=1}^{N_{S}}\left[I_{S}^{\left(j\right)}-\frac{1}{N_{S}}\sum_{j=1}^{N_{S}}I_{S}^{\left(j\right)}\right]}}$$

where $I_{D}^{\left(i\right)}$ and $I_{S}^{\left(j\right)}$ represent the grayscale value of the *i*-th pixel in the defective area and the *j*-th pixel in the non-defective area, respectively, and $N_{D}$ and $N_{S}$ represent the number of pixels in the defective and non-defective area respectively. 

**Figure 7:**
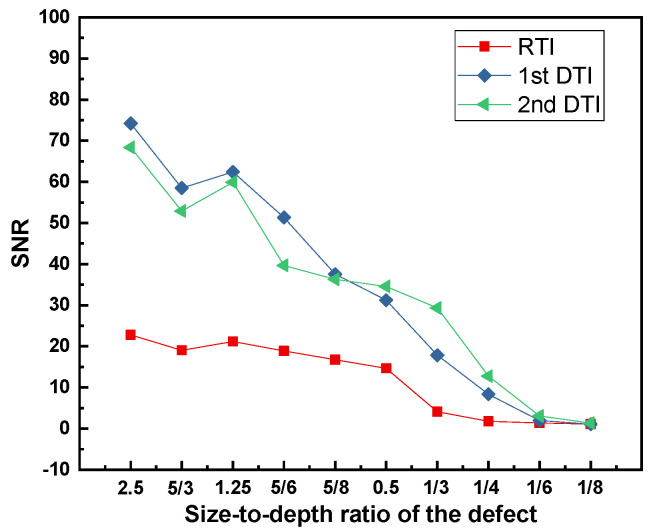
The SNR for each size-to-depth ratio of the defect.

The SNR for each size-to-depth ratio of the defect by employing different processing approaches has been analyzed as shown in Figure 7. This part of the analysis can provide a reference for processing defects with different size-to-depth ratios using different processing methods.

In the first and second order DTIs, the contrast between defective and sound areas was significantly improved and more defects can be recognized. It is noteworthy that the grayscale of defects is higher than the background in the first stage, and then becomes lower in later frames. This phenomenon reveals that the contrast between defective and sound areas in DTI changes from positive to negative, which has been theoretically demonstrated in Section 2. The relationship between the defect depth and its display time still holds. In other words, the deeper the defect, the later it can be found in DTI. The defect C1, which is 8 mm deep from the surface, began to be observed (the grayscale became higher than the background) in Figure 6d,f, while the grayscale of defects As and Bs turned lower than the background at the same time.

**Figure 8:**
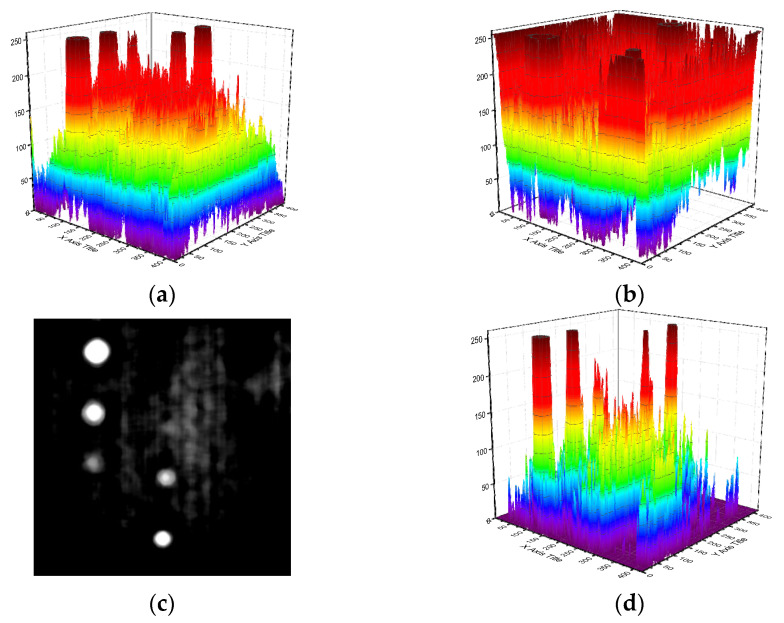
Detection enhancement of FDTI on voids in silicone rubber. (**a**) SNR of 10th frame 1st DTI. (**b**) SNR of 275th frame 1st DTI. (**c**) FDTI from subtracting 1st DTIs. (**d**) SNR of FDTI.

For further explanation, the signal intensity representing the grayscale of pixels is presented in Figure 8. Figure 8c shows the FDTI obtained by subtracting the 10th and 275th frame 1st DTI, which are before and after the grayscale inversion, respectively. From the comparison of Figure 8a,b,d, the identification of defects in FDTI is well enhanced while the SNR is truly improved in FDTI.

### 4.2. Detection of Cracks in Fiber-Reinforced Plastic

**Figure 9:**
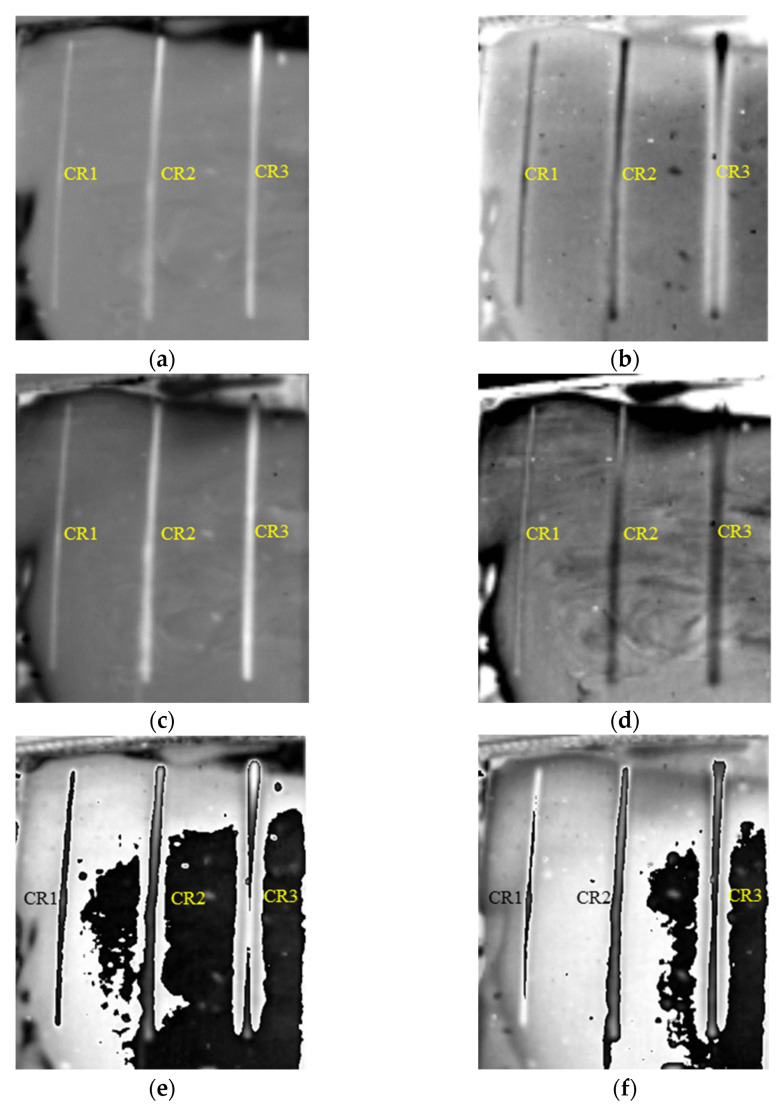
Detection results of cracks in FRP bonded with 1 mm thick SIR. (**a**) The 628th frame 1st DTI. (**b**) The 819th frame 1st DTI. (**c**) The 589th frame 2nd DTI. (**d**) The 829th frame 2nd DTI. (**e**) FDTI from subtracting 1st DTIs. (**f**) FDTI from subtracting 2nd DTIs.

As shown in Figure 9, all three cracks (CR) below 1 mm thick SIR can be clearly identified in both the first and second order DTIs. The defect identification is further improved by subtracting the two 1st and 2nd DTIs respectively before and after the grayscale inversion as shown in Figure 9e,f.

**Figure 10:**
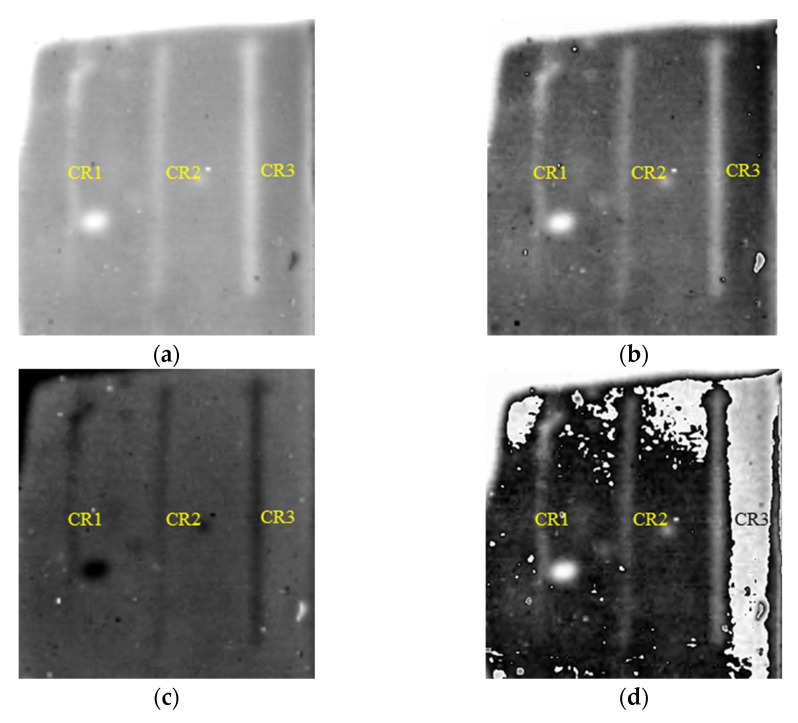
Detection results of cracks in FRP bonded with 3 mm thick SIR. (**a**) The 1570th frame 1st DTI. (**b**) The 1479th frame 2nd DTI. (**c**) The 2487th frame 2nd DTI. (**d**) FDTI from subtracting 2nd DTIs.

**Figure 11:**
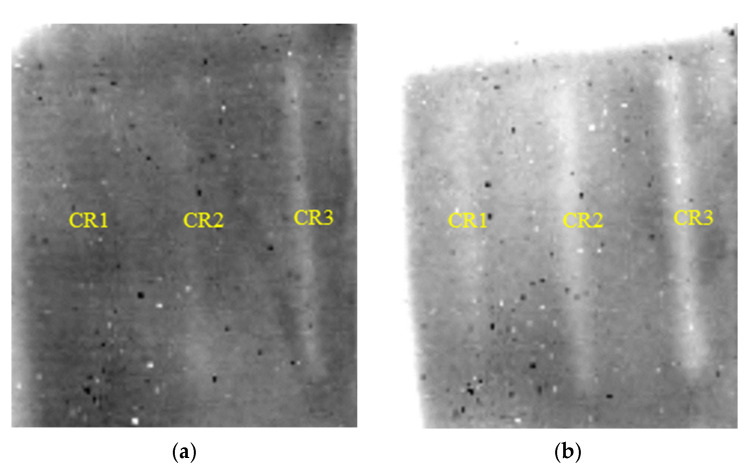
Detection results of cracks in FRP bonded with 5 mm thick SIR. (**a**) The 1114th frame 1st DTI. (**b**) The 2947th frame 2nd DTI.

Figure 10 shows the detection results of cracks in FRP bonded with 3 mm thick SIR. Similarly, the contrast between defective and sound areas FDTI is enhanced. The first and second order DTIs revealing cracks in FRP bonded with 5 mm thick SIR are presented in Figure 11. It is concluded that cracks with a width of 0.5 mm below 5 mm thick SIR can be tested at least.

### 4.3. Detection of Disbond in SIR–FRP Bonding Structure

**Figure 12:**
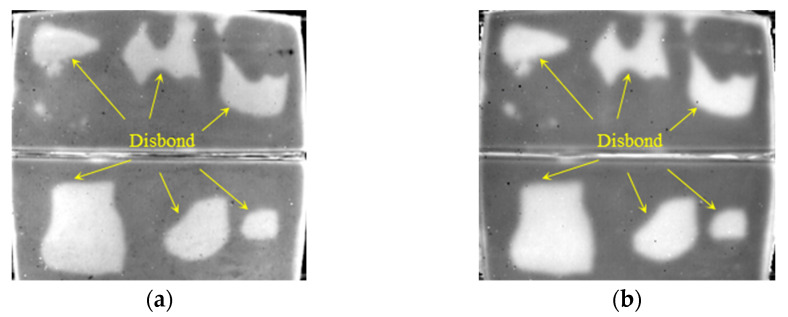
Detection results of disbond with 2 mm thick SIR. (**a**) The 829th frame 1st DTI. (**b**) The 1144th frame 2nd DTI.

**Figure 13:**
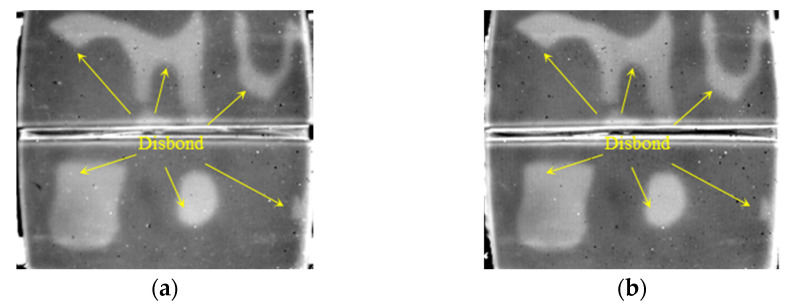
Detection results of disbond with 3 mm thick SIR. (**a**) The 1898th frame 1st DTI. (**b**) The 1046th frame 2nd DTI.

**Figure 14:**
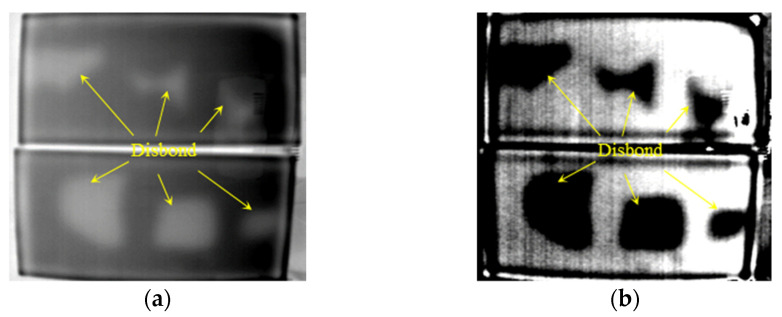
Detection results of disbond with 5 mm thick SIR. (**a**) The 2117th frame 1st DTI. (**b**) The 3476th frame 2nd DTI.

The first and second order DTIs of SIR–FRP bonding structure of 2 mm, 3 mm, and 5 mm thick SIR are illustrated in Figure 12, Figure 13 and Figure 14, where all the disbond defects can be easily recognized. It is concluded that interface disbond of SIR–FRP bonding structure below 5 mm thick SIR can be tested at least.

## 5. Conclusions

In this work, AIT was employed to detect various types of defects in SIR–FRP bonding structure in order to verify its feasibility for defect detection of composite insulating components in power equipment. Experiments on defective SIR–FRP plate specimens were performed, and the results show that void, crack, and interface disbond of certain sizes and depths in SIR–FRP structure can be easily detected with the suggested method. Logarithm operation and polynomial fitting were used to reconstruct thermal image sequence for reducing the amount of data to be stored and the interframe noise in time domain. The effectiveness of differential thermal image for enhancing the contrast of defective and sound areas and eliminating the influence of additive noise and uneven heating was verified via theoretical analysis and experiments. It is noteworthy that frame difference thermal image obtained by subtracting two differential thermal images extracting from respectively before and after the contrast inversion was proposed to enhance defect identification. 

Compared with other existing methods, the PT method studied in this paper shows some great advantages. All the defects of specimens showed up within the acquisition time of 30 s. The detection process is short and the detection area is large each time, which ensures fast detection, intuitive results, and simple operation. As demonstrated, the detection accuracy of PT can be further enhanced via proposed image processing techniques. Last but not the least, the proposed method is immune to electromagnetic interference and has the potential to perform detection at long distances. Therefore, PT would be a promising approach for live detection of power equipment. For this purpose, the following studies will be carried out in the future:

(1) The influence of field operation conditions, including contamination, abnormal temperature rise, different measurement distance determined by the voltage class, etc., on the detection results.
(2) The influence of excitation and acquisition process settings on the detection results.
(3) Advanced image processing techniques for further improving the detection capability of active infrared thermography.

## Data Availability

The data that support the findings of this study are available from the corresponding author upon reasonable request.
